# Achieving High Strength and Creep Resistance in Inconel 740H Superalloy through Wire-Arc Additive Manufacturing and Thermodynamic-Guided Heat Treatment

**DOI:** 10.3390/ma16196388

**Published:** 2023-09-25

**Authors:** Soumya Sridar, Luis Fernando Ladinos Pizano, Michael A. Klecka, Wei Xiong

**Affiliations:** 1 Physical Metallurgy and Materials Design Laboratory, Department of Mechanical Engineering and Materials Science, University of Pittsburgh, Pittsburgh, PA 15261, USA; sos57@pitt.edu (S.S.); lul58@pitt.edu (L.F.L.P.); 2 RTX Technology Research Center, 411, Silver Lane, East Hartford, CT 06118, USA; michael.klecka@rtx.com

**Keywords:** CALPHAD, recrystallization, stress rupture, anisotropy, diffusion

## Abstract

Inconel 740H superalloy is commonly used in advanced ultra-supercritical power plants since it possesses excellent strength and creep resistance. This study investigates the microstructure and mechanical properties of Inconel 740H superalloy fabricated using wire-arc additive manufacturing. The as-printed microstructure consisted of columnar γ grains with the Laves phase and (Nb, Ti)C carbides as secondary phases. The anisotropy in grain structure increased from the bottom to the top regions, while the hardness was highest in the middle portion of the build. To guide the post-heat treatment design, thermodynamic and kinetic simulations were employed to predict the temperature and time. Complete recrystallization with the Laves phase dissolution occurred throughout the build after homogenization at 1200 °C for 2 h. The peak hardness was achieved after aging at 760 °C for 12 h with the M_23_C_6_ carbides decorating the grain boundaries and γ’ precipitates in the grain interior. The yield strength (655 MPa) and ductility (29.5%) in the post-heat treated condition exceeded the design targets (620 MPa, 20%). Stress rupture tests at 750 °C showed that the high-temperature performance was at par with the wrought counterparts. The fracture mode after rupture was identified to be intergranular with the presence of grain boundary cavities along with grain boundary sliding.

## 1. Introduction

Inconel 740H is a γ’ strengthened Ni-base superalloy developed by Special Metals Corporation [1], which is primarily used in advanced ultra-supercritical (AUSC) power plants as superheater and reheater tubes that are subjected to high pressure (35 to 45 MPa) and temperatures upto 800 °C. This material is suitable for high-temperature applications owing to its excellent creep and oxidation resistance, as well as microstructure stability and high strength at elevated temperatures [2]. This alloy is derived from its predecessor, Inconel 740, with adjustments in the Al/Ti ratio to avoid the formation of a deleterious η (Ni_3_Ti) phase during service [3]. Moreover, the reduction in the silicon, boron, and niobium content in Inconel 740H compared to the parent alloy maximized the weldability by decreasing the susceptibility to liquation cracking [4]. This alloy has been considered a suitable candidate for several power generation applications, such as thermal power plants with super-critical CO_2_ [5] and concentrated solar power plants [6]. Inconel 740H in its wrought form has been investigated to determine several critical properties such as corrosion, oxidation, and fatigue resistance for its use in these applications [7,8,9,10].

To the best of our knowledge, no reports are available in the literature for the fabrication of monolithic Inconel 740H superalloy by additive manufacturing. Since this alloy possesses superior weldability and is available in wire form, it is a potential candidate to be fabricated using wire-arc additive manufacturing (WAAM). WAAM is a directed energy deposition (DED) based additive manufacturing technique that uses a plasma or electric arc to melt the wire feedstock for layer-by-layer deposition. This technique is capable of fabricating large-sized components due to its high deposition rate and heat input. The material costs are lesser than any powder-based DED process as the wire feedstock is cheaper than metal powders [11]. However, the microstructure in the as-printed condition might not be suitable for high-temperature applications due to the anisotropy in the matrix due to the columnar grains and lack of adequate strengthening precipitates, which necessitates the design of a post-heat treatment. The standard post-heat treatment for wrought Inconel 740H superalloy is homogenization at 1150 °C for 30 min followed by water quenching and aging at 800 °C for 16 h [3]. The mechanical properties reported for wrought Inconel 740H are yield strength of 724 MPa, ultimate tensile strength of 1066 MPa, and elongation of 28.1% [12]. Though a standard post-heat treatment is available for traditionally manufactured Inconel 740H superalloys, it may not be suitable to apply them for additively manufactured alloys due to the microstructure complexity owing to heating and cooling cycles experienced during deposition as it has been demonstrated for several other additively manufactured alloys [13,14].

**Figure 1:**
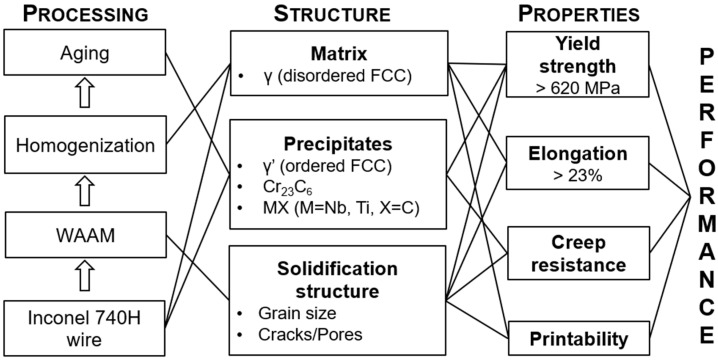
Systems design chart for fabrication of Inconel 740H superalloy using wire-arc additive manufacturing along with post-heat treatment. The microstructural features and desired mechanical properties are listed.

To assess the feasibility of fabricating Inconel 740H superalloy using WAAM along with the design of post-heat treatment, a systems design chart [15] was formulated that links the processing steps, macro/microstructure, and target properties for good performance, as shown in Figure 1. The effect of each processing step on the microstructure and the influence of this resultant structure on the mechanical properties, as well as the printability, is depicted concisely in this chart. A two-step post-heat treatment consisting of homogenization to recrystallize the microstructure, relieve the residual stresses, and dissolve the secondary phases, followed by aging to precipitate the strengthening phases is essential for improving the properties. The intended microstructure of Inconel 740H superalloy fabricated using WAAM after post-heat treatment will be a γ matrix consisting of γ’ precipitates for strengthening and continuous M_23_C_6_ carbides along the grain boundaries to enhance the creep resistance. MX (M = Ti, Nb; X = C, N) phase will act as pinning particles to avoid excessive grain coarsening during homogenization. It is to be noted that the design targets for the tensile properties were obtained from the ASME Code Case 2702, which certifies Inconel 740H superalloys for service in AUSC power plants in 2011 [16].

The fabrication of Inconel 740H superalloy using WAAM has been explored for the first time in this work. Since WAAM is capable of fabricating large builds, there is a need to study the variation in the microstructure and properties along the build direction due to the difference in thermal history for the as-printed Inconel 740H superalloy. Thermodynamic and kinetic simulations guided the design of the post-heat treatment, and its efficacy was determined by evaluating the tensile properties in as-built and post-heat treated conditions. This work will provide an understanding of the applicability of WAAM for fabricating large builds of Inconel 740H superalloy and the mitigation measures required for improving the mechanical properties.

## 2. Materials and Methods

### 2.1. Experimental Details

**Figure 2:**
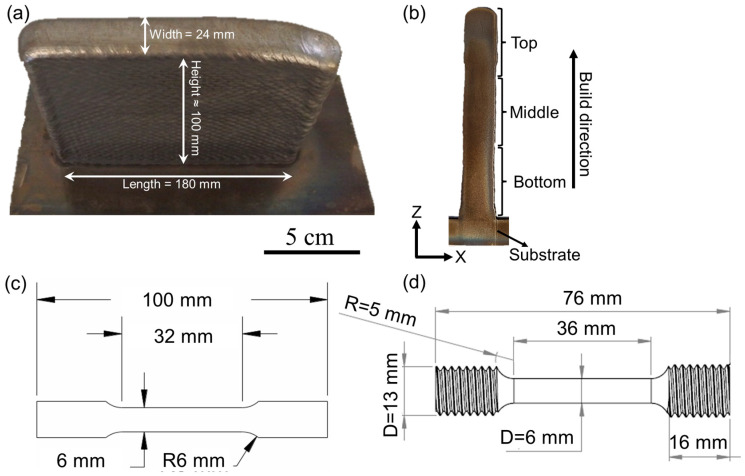
(**a**) Final Inconel 740H superalloy build fabricated using WAAM with the dimensions marked, (**b**) A slice of XZ plane extracted from the final build showing the different portions into which the sample was partitioned to study the variation in properties along the build direction and schematic of the sample used for (**c**) tensile and (**d**) stress rupture tests with its dimensions.

The composition (in wt.%) of Inconel 740H (denoted as 740H hereafter) superalloy wire with 1 mm diameter as specified by the vendor (Special Metals Welding Products Co., Huntington, WV, USA) is listed in Table 1. The sample was built over a mild steel substrate in Raytheon Technologies Development Centre with an ABB robotic WAAM system with a plasma torch connected to a six-axis robot. An average voltage of 20.9 V, layer height of 3 mm, and wire feed rate of 2.5 m/min was maintained throughout the deposition of 50 layers of 740H superalloy. Argon gas with a flow rate of 1.2 L/min was used for producing plasma and shielding the welding torch. The wire feed angle was maintained as 30° and a 12 mm distance was maintained between the torch and the build. An overall deposition rate of 0.9 kg/h was maintained throughout the deposition. The substrate temperature was maintained at 100 °C before the start of the deposition. A higher current (220 and 200 A) and lower travel speed (3.5 and 4 mm/s) was used for the first and second layers to ensure good adhesion with the substrate. Subsequently, a constant current of 166 A and travel speed of 5 mm/s was employed from the 3rd to 50th layer to complete the build. A square raster or lawnmower-type fill pattern with a width of 16 mm and step-over of 3 mm was used for the deposition of all the layers to achieve high stability of the melt pool without any bulging, sagging, or overflow. The dimensions of the final deposit are 180 mm in length, 24 mm in width, and approximately 100 mm in height, as shown in Figure 2a. A two-step post-heat treatment consisting of homogenization and aging steps was designed to improve the mechanical performance of the WAAM 740H superalloy. The homogenization temperature and time were designed based on thermodynamic and kinetic simulations (described in Section 2.2). After the application of the designed homogenization heat treatment, aging time was further optimized with the aging temperature identified from thermodynamic calculations.

The builds deposited using WAAM were sectioned along the build direction using electric discharge machining (Mitsubishi MV2400S, Tatebayashi, Japan) to view the XZ plane for further characterization. As shown in Figure 2b, the extracted sample was divided into three regions namely, top, middle, and bottom to investigate the variation in properties at different locations along the build direction. The sample surfaces were mechanically ground from 600 to 1200 grit emery papers and polished using diamond (3 and 1 μm particles) and silica (0.04 μm particles) suspensions. To reveal the precipitates that formed after aging heat treatment and stress rupture testing, electrolytic etching was carried out using chromic acid (H_2_CrO_4_) prepared from 15 g of chromium oxide (CrO_3_) and 10 mL of concentrated sulfuric acid (H_2_SO_4_) dissolved in 150 mL of 85% phosphoric acid (H_3_PO_4_) at a voltage of 5 V for 20 s. The as-polished and etched surface was viewed under FEI Scios Dual Beam focused ion beam—scanning electron microscope (SEM) attached with a field emission gun source. The elemental composition was determined using the OctaneElite energy dispersive spectroscope (EDS) system. The grain structure analysis was carried out using electron backscattered diffraction (EBSD) with a EDAX Hikari EBSD camera and analyzed using the TSL-OIM software (version 8).

Hardness measurements were carried out using the automated Vickers microhardness tester (AMH55 with LM310AT Microindenter, LECO Corporation, St. Joseph, MI, USA) with a 300 g load and 10 s dwell time. For generating the hardness maps, ~600 indentations were performed in the top, middle, and bottom portions of the sample. For determining the hardness after aging heat treatment, an average of 20 hardness readings was considered. The room temperature tensile tests were carried out using a 500 kN servo-mechanical load frame with a crosshead speed of 1.3 mm/min with the loading direction parallel to the build direction. Flat dog bone-shaped samples were machined with dimensions according to the ASTM E8 standard [17] from the build, as shown in Figure 2c, and three tests were performed for as-built and post-heat treated conditions. Each sample was marked with reflective tags such that the elongation could be monitored in a non-contact mode using a laser-based extensometer during the test. Stress rupture tests were performed at 750 °C with applied stresses of 375, 400, 425, and 450 MPa for the bars extracted from the WAAM builds with dimensions shown in Figure 2d.

### 2.2. Computational Details

**Figure 3:**
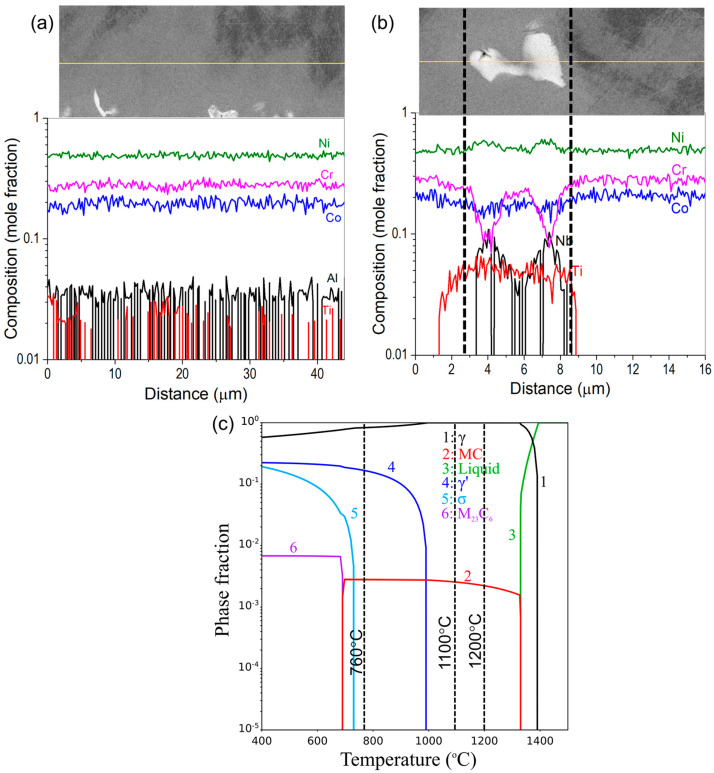
(**a**) Composition of the matrix in the interdendritic region showing negligible composition inhomogeneity, (**b**) Composition of the matrix with the Laves phase determined using EDS which will serve as input for the DICTRA simulations (yellow line indicates the line along with the EDS scan was performed) and (**c**) Calculated equilibrium phase fractions as a function of temperature for the 740H superalloy wire composition to identify the homogenization and aging temperatures for post-heat treatment.

The kinetic simulations were performed using the DICTRA module of Thermo-Calc software (version 2021b) with TCNI8 and MOBNI4 as thermodynamic and mobility databases, respectively. EDS line scan was performed in the region with the Laves phase in the γ (FCC) matrix of the as-built 740H superalloy to obtain the required inputs for the DICTRA simulations. Two different areas in the matrix, namely, the interdendritic regions and the regions with the Laves phase were probed using line EDS to identify the region with maximum inhomogeneity (Figure 3a,b). Based on the composition variation as a function of distance obtained from the EDS line scan, it was found that the maximum inhomogeneity in composition occurs in the region of the matrix containing the Laves phase. Therefore, it was considered as input for obtaining the optimum homogenization temperature and time using the kinetic simulations. The calculated equilibrium phase fraction as a function of temperature for the 740H superalloy wire composition using Thermo-Calc is shown in Figure 3c. This calculation was performed using the commercial multicomponent thermodynamic database for Ni-based superalloys namely, TCNI8, available in the Thermo-Calc software (version 2021b). From this plot, it was identified that the homogenization temperature can be either 1100 or 1200 °C due to the absence of deleterious secondary phases, and hence, kinetic calculations were performed for both temperatures. The presence of NbC particles at these temperatures is not considered deleterious since they act as pinning particles to arrest the grain growth during the homogenization heat treatment.

To design the homogenization heat treatment, CALPHAD-based kinetic simulations were performed using the DICTRA module of Thermo-Calc software (version 2021b) to predict the time required to dissolve the Laves phase into the matrix. The simulation cell was considered as a single-phase FCC where the Laves phase region was considered as an FCC phase with higher Nb and Ti content rather than incorporating a two-phase moving boundary problem with FCC and Laves phases described separately to model the dissolution. The moving boundary problem could not be implemented due to the lack of reliable mobility expressions for the Laves phase in the commercial mobility database (MOBNI4) and in the literature. Hence, a single-phase FCC was considered, which was found to estimate the homogenization time closer to the experimental value for the Inconel 718 superalloy, as reported in our previous work [18]. The simulations were performed for 10^6^ s with 200 grid points. Additional inputs such as the width of the cell (total length of the line EDS), number and position of each grid point within the cell (the total number of points and spacing between them in the line EDS), and composition at each grid point (composition at each point of the line EDS) were chosen from the line scan obtained using EDS.

## 3. Results and Discussion

### 3.1. As-Built WAAM Inconel 740H Superalloy

**Figure 4:**
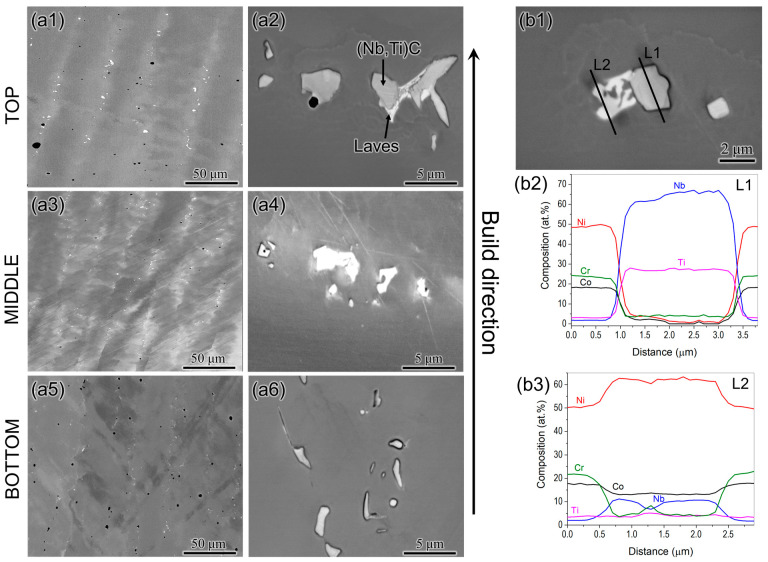
SEM micrographs from (**a1**,**a2**) top, (**a3**,**a4**) middle and (**a5**,**a6**) bottom portions of the WAAM 740H superalloy build in as -built condition showing a γ matrix with columnar grains and secondary phases such as the Laves phase and (Nb, Ti)C and (**b1**–**b3**) SEM micrograph of the second phases in the build and the corresponding EDS line scans confirming their composition. Li corresponds to (Nb,Ti)C phase and L2 denotes the Laves phase rich in Nb.

Since a large build of Inconel 740H superalloy was fabricated using WAAM in this work, the sample needs to be investigated in different regions to understand the location-specific variation in properties along the build direction in as-built condition. No visible cracks were found in the build, owing to the excellent weldability of the 740H superalloy. The SEM micrographs were obtained in backscattered electron mode from the top, middle, and bottom portions of the sample, as shown in Figure 4(a1–a6). A matrix with a columnar grain structure with secondary phases, such as the Laves phase and (Nb, Ti)C precipitates can be observed. The composition of the secondary phases were confirmed using the EDS line scans (Figure 4(b1–b3)) and also indicating that the Laves phase is rich in Nb. The Laves phase was predominantly found in the interdendritic region, indicating the strong segregation of Nb and Ti in these regions during the solidification. It is also to be noted that several gas pores in spherical shape can be noticed throughout the sample. The possible source for the formation of gas pores could be the dissolution of the shielding gas during the deposition. The average fraction of gas pores was calculated to be 0.32% using ImageJ software (version 1.54f) from the SEM micrographs.

**Figure 5:**
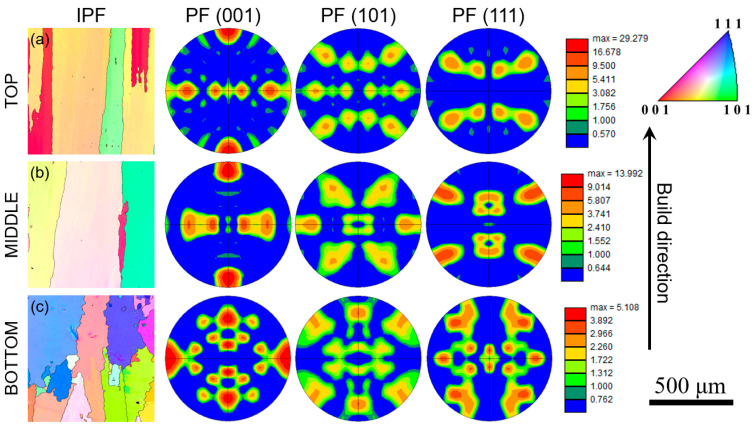
Inverse pole figure and pole figure intensity maps for (001), (101) and (111) planes obtained using electron backscattered diffraction from (**a**) top, (**b**) middle and (**c**) bottom portions of the WAAM 740H superalloy build in as-built condition showing the variation in anisotropy along the build direction.

Figure 5 shows the inverse pole figure (IPF) maps and pole figure (PF) intensity maps for (001), (101), and (111) planes obtained using EBSD from the top, middle, and bottom portions of the sample. The grains in the bottom region are found to be less columnar in comparison with the top and middle portions of the sample. This is because the bottom region of the build is close to the mild steel substrate with a higher thermal conductivity than the 740H superalloy, and hence, the substrate acts as an efficient heat sink to dissipate the heat from the deposited material. On the contrary, in the top and middle portions of the build with higher deposition heights, the previously deposited 740H superalloy with lower thermal conductivity acts as the substrate for the freshly deposited material, and hence, the heat dissipation will be slower, leading to the formation of columnar grains. From the maximum intensity of the PF intensity maps for different planes, it is evident that the degree of anisotropy increases from the bottom to the top portion of the sample. A higher maximum intensity indicated higher anisotropy, which is found in the top portion of the sample, and the grains are mostly oriented in the (001) planes, as observed from the PF intensity maps. Due to the high heat input in WAAM, the deposition of a fresh layer leads to remelting of the already solidified layers beneath it. This leads to the same grain growing over several layers with identical orientations leading to anisotropy because of the inhomogeneous heat dissipation triggered by the migrating arc during the deposition [19].

**Figure 6:**
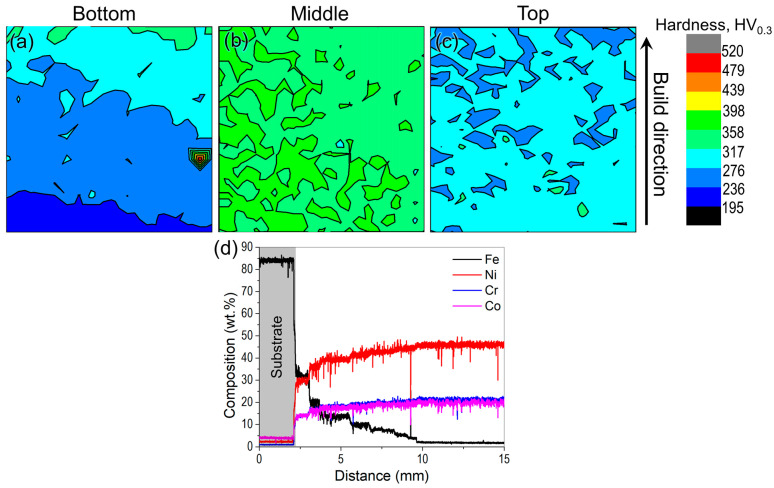
(**a**) Microhardness maps obtained from the (**a**) top, (**b**) middle and (**c**) bottom portions of the WAAM 740H superalloy build in as-built condition showing the drastic variation in microhardness in different regions along the build direction (area for each microhardness map is 3 × 3 cm^2^) and (**d**) Composition as a function of distance from the substrate to the bottom portion of the build showing the diffusion of Fe into the 740H superalloy build for ~5 mm height of the build.

The microhardness maps from different regions of the sample are shown in Figure 6a. In the bottom portion of the sample, a region with very low hardness (195–275 HV_0.3_) was identified, which forms due to the diffusion of iron from the mild steel substrate, which was confirmed using an EDS line scan as shown in Figure 6b. The diffusion is found to be rapid as the effect of the Fe diffusion on the hardness can be found up to nearly 5 mm away from the substrate. This suggests that the compatibility between the substrate and alloy to be deposited using WAAM is important, and a Ni alloy substrate can be employed to avoid the Fe diffusion in the 740H superalloy. Moreover, the hardness in the middle portion (320–400 HV_0.3_) was higher than the top portion (240–275 HV_0.3_) of the sample. This is because as the deposition progresses, heat accumulation occurs in the building. Since the top portion is farther away from the mild steel substrate, the heat transfer must happen through the previously deposited layers that will be slower, leading to grain coarsening with slower cooling rates and reduced hardness [20]. Accordingly, it has been observed from the grain structure analysis using EBSD (Figure 5) that the average grain size in the top portion of the build is 405 μm whereas the corresponding value in the bottom portion is 285 μm indicating coarsening of grains in the top portion of the build. It has also been reported that remelting during WAAM leads to inhomogeneous heat dissipation with increased nucleation rate and reduced growth rate, and this results in fine grains forming along the melt pool boundaries [19]. Since the middle portion experiences a higher number of remelting and solidification cycles when compared to the top region, the finer grains at the melt pool boundaries result in higher average hardness. With this observation, the middle portion is expected to possess higher residual stresses compared to the top and bottom regions.

### 3.2. Post-Heat Treatment Design for WAAM Inconel 740H Superalloy

A two-step post-treatment will be designed for Inconel 740H superalloy fabricated using WAAM due to the microstructural heterogeneities in the as-built condition as elaborated in the previous sub-section. The anisotropic grains in the as-built WAAM 740H superalloy need to be recrystallized to obtain an isotropic equiaxed matrix, and the secondary precipitates, such as the Laves phase need to be dissolved into the matrix using the homogenization step. The aging step is required to induce the precipitation of strengthening phases, such as γ’, along with M_23_C_6_, to improve the mechanical performance. The standard heat treatment for Inconel 740H superalloy, as recommended by Special Metals Co., is homogenization above 1100 °C followed by air cooling or water quenching and aging in the temperature range of 760–816 °C for longer than 4 h [1]. The common practice for wrought Inconel 740H superalloy is homogenization at 1150 °C for 30 min, followed by water quenching and aging at 800 °C for 16 h [3].

#### 3.2.1. Homogenization

**Figure 7:**
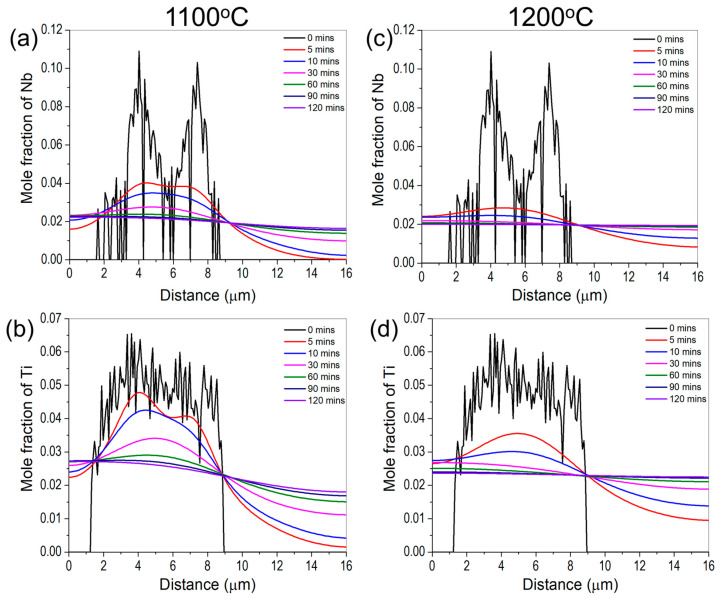
Calculated variation in composition of Nb and Ti as a function of distance for different times obtained from DICTRA at (**a**,**b**) 1100 °C and (**c**,**d**) 1200 °C showing the flattening of composition corresponding to the dissolution of the Laves phase.

As mentioned earlier, the homogenization temperatures were chosen based on the equilibrium phase fraction vs. temperature plot for the wire feedstock composition in Figure 3b. Since the standard heat treatment for wrought Inconel 740H superalloy needs homogenization at 1150 °C for 30 min, we chose the homogenization temperature around the standard heat treatment temperature to deduce the optimum time to avoid deviating excessively from it. The change in composition of Nb and Ti as a function of time obtained from the DICTRA simulations at 1100 and 1200 °C are shown in Figure 7. The flattening of the composition indicates that the precipitate has dissolved into the matrix. It is evident from this figure that the composition of Nb and Ti almost flattens close to 60 and 30 min at 1100 and 1200 °C, respectively. It is to be noted that the predicted time is only for dissolving the Laves phase into the matrix, however, recrystallization of microstructure is additionally required during homogenization.

**Figure 8:**
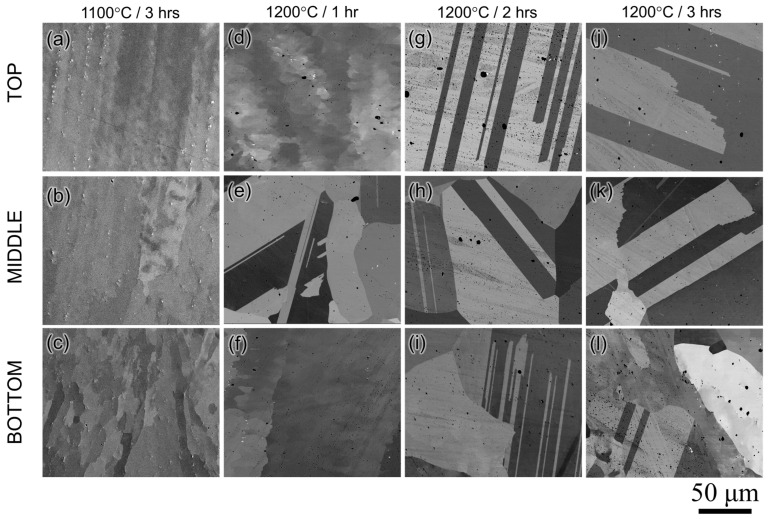
SEM micrographs from the top, middle and bottom regions of the sample homogenized at 1100 °C for (**a**–**c**) 3 h and 1200 °C for (**d**–**f**) 1 h, (**g**–**i**) 2 h and (**j**–**l**) 3 h showing complete recrystallization along with the formation of annealing twins for samples homogenized at 1200 °C for 2 and 3 h.

Therefore, the homogenization heat treatment for the as-built WAAM 740H superalloy was performed at 1100 and 1200 °C for 1, 2, and 3 h, followed by water quenching. The SEM micrographs from the top, middle, and bottom portions of the samples homogenized at 1100 and 1200 °C for different times are shown in Figure 8. The homogenization heat treatments were performed at 1100 °C for 1, 2, and 3 h, however, it was found that the sample did not recrystallize completely, even after 3 h. The microstructure, after homogenization at 1100 °C for 3 h, retained the columnar microstructure (Figure 8a–c), indicating that this temperature is not sufficient to recrystallize the microstructure throughout the build. After homogenization for 1 h at 1200 °C, the matrix was recrystallized only in the middle portion and still retains the columnar grain structure in the top and bottom portions of the sample. However, no dominant Laves phase was found in the matrix, indicating its complete dissolution. This proves that the predicted homogenization time from the kinetic simulations is in good agreement with the experimental observations. It is also interesting to note that the microstructure in the middle region gets recrystallized faster than the top and bottom portions. This can be correlated with the higher residual stress in the middle region leading to higher hardness as observed from the hardness maps for the as-built WAAM 740H superalloy (Figure 6b). Since residual stresses are the major driving force for recrystallization, the higher residual stress in the middle portion promotes faster recrystallization than other regions. After homogenization at 1200 °C for 2 and 3 h, the matrix is completely recrystallized throughout the build accompanied by the formation of annealing twins (Figure 8g–l). It can be found that the grain size and annealing twins in the sample homogenized for 3 h are coarser than the 2 h homogenized sample. After homogenization at 1200 °C for 2 h, the average grain size was measured to be 600 μm while the corresponding value in as-built condition is 380 μm. Therefore, to avoid further grain coarsening after recrystallization, the optimum homogenization time was determined to be 2 h at 1200 °C.

#### 3.2.2. Aging

**Figure 9:**
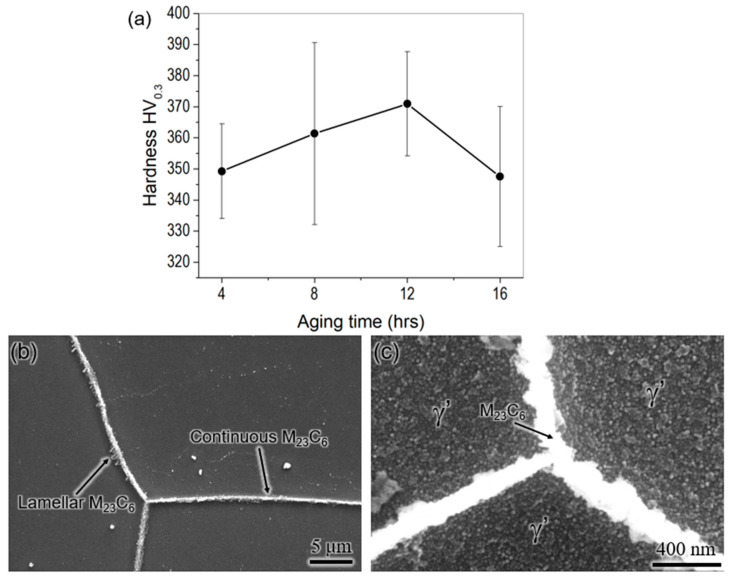
(**a**) Microhardness of aged WAAM 740H superalloy as function of aging time showing the peak hardness being achieved after 12 h of aging at 760 °C and (**b**,**c**) SEM micrographs from WAAM 740H superalloy aged at 760 °C for 12 h after etching indicating the presence of continuous and lamellar M_23_C_6_ in the grain boundaries and γ’ phase in the grain interior.

The aging temperature for WAAM 740H superalloy was identified as 760 °C, which is slightly lesser than the aging schedule from the standard heat treatment for wrought Inconel 740H superalloy, which is 800 °C for 16 h. This aging temperature was chosen for two reasons. Firstly, at 760 °C the maximum γ’ phase fraction was predicted according to the equilibrium phase fraction vs. temperature plot (Figure 3b). Secondly, this work is part of a project proposed for joining P91 steel with 740H superalloy using WAAM, and hence, we wanted to ensure the same post-heat treatments for both materials. Since aging at 800 °C leads to the formation of α-ferrite in P91 steel, the aging temperature was reduced by 40 °C for better compatibility. The aging heat treatment was performed after homogenization at 1200 °C for 2 h. The microhardness as a function of aging time (Figure 9a) shows that the peak hardness is achieved after aging at 760 °C for 12 h. The optimum aging time is found to be 4 h less than the standard aging time of 16 h, even though the standard aging temperature is higher than that chosen in this work. This observation can be attributed to the effective homogenization of the matrix at higher temperatures and longer time for WAAM 740H superalloy (1200 °C/2 h) compared to standard homogenization (1150 °C/30 min). The designed homogenization heat treatment has effectively dissolved the secondary phase, leading to higher supersaturation in the matrix, and hence, peak aging is achieved with shorter aging time at lower temperatures when compared to the standard aging heat treatment (800 °C/16 h).

Figure 9b,c show the SEM micrographs of WAAM 740H superalloy aged at 760 °C for 12 h after etching. The grain boundaries were decorated with continuous and thin M_23_C_6_ precipitates, along with few lamellar M_23_C_6_ precipitates protruding away from the grain boundary. Moreover, the grain interior was completely filled with the γ’ precipitates, which will lead to the strengthening of the material. Both phases are important for achieving excellent creep resistance in Inconel 740H superalloy, which has been precipitated in desired morphology using the post-heat treatment. The γ’ size was not measured from the SEM micrographs since it could not be resolved clearly. Therefore, an optimum post-heat treatment has been designed for WAAM 740H superalloy with homogenization at 1200 °C for 2 h and aging at 760 °C for 12 h. Its effectiveness will be evaluated by evaluating the mechanical properties, as explained in the upcoming section.

### 3.3. Mechanical Properties of WAAM Inconel 740H Superalloy

**Figure 10:**
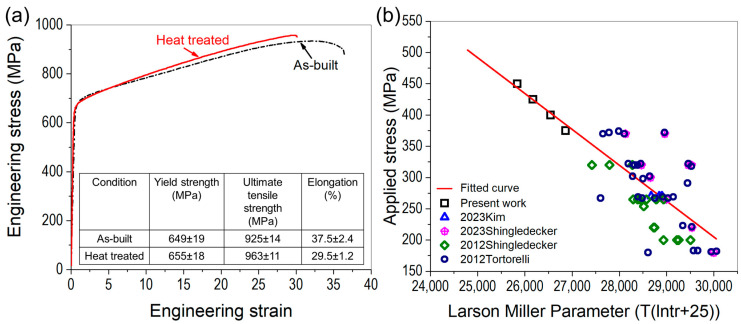
(**a**) Engineering stress-strain curves for WAAM 740H superalloy in as-built and post-heat treated conditions along the list of tensile properties compared with the design targets and (**b**) Larson-Miller parameter vs applied stress curve for the stress rupture performance showing that the post-heat treated WAAM 740H superalloy is at par with its wrought counterparts reported in literature.

**Figure 11:**
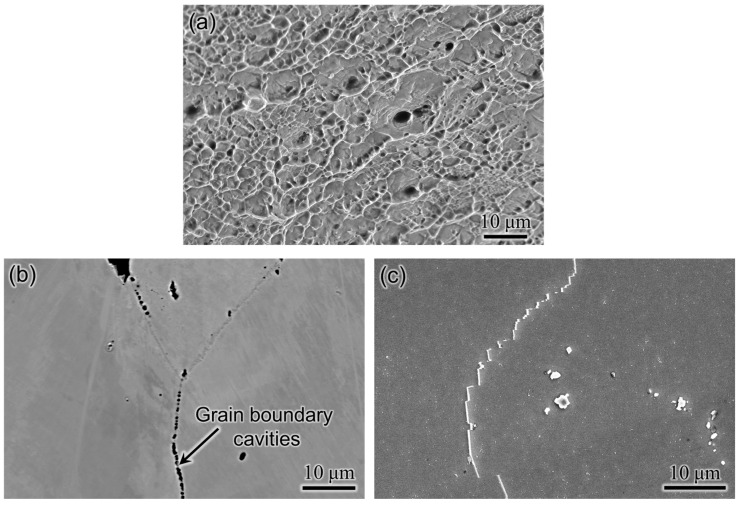
(**a**) Fractography after tensile testing for post-heat treated WAAM 740H superalloy showing a ductile mode of fracture with the presence of dimples along with small gas pores. SEM micrographs after stress rupture tests for WAAM 740H superalloy; (**b**) Grain boundary cavities close to the fracture surface indicating an intergranular mode of fracture and (**c**) Serrated grain boundaries showing grain boundary sliding during the application of stress at high-temperature.

The engineering stress-strain curves, along with the tensile properties of WAAM 740H superalloy, in as-built and post-heat-treated conditions are shown in Figure 10a. The yield strength increases, and consequently, the ductility decreases with the application of the designed post-heat treatment. These quantities are well above the target in both as-built and post-heat treated conditions. However, the yield strength obtained in this work is lower than the corresponding values obtained for wrought 740H superalloy (~724 MPa) reported by Special Metals Co [1]. This can be attributed to the presence of gas pores throughout the build in the as-built condition and after applying the homogenization heat treatment. The fracture surface after the tensile test for post-heat treated WAAM 740H superalloy was observed using SEM, as shown in Figure 11a. It can be found that a ductile mode of fracture has occurred, which is confirmed by the presence of the dimples whereas small pores were also present, which could be gas pores. It has been reported that micropores and undetectable inhomogeneities in as-built conditions can coalesce to reduce the surface energy during the heat treatment to increase the porosity [21]. This emphasizes the application of hot isostatic pressing (HIP) to promote pore closure along with recrystallization of the microstructure. There are reports available in the literature that highlight the importance of HIP in improving the mechanical properties of alloys processed using different additive manufacturing techniques [22,23,24]. Hence, to improve the mechanical properties of WAAM 740H superalloy, HIP is necessary since the traditional furnace heat treatment without applied external pressure cannot induce pore closure. Another possible method to coalesce the gas pores is by fabricating Inconel 740H superalloy using hot forging WAAM [25], which is a new variant of WAAM that is capable of closing the pores during the deposition.

Stress rupture tests were performed under different stresses (375, 400, 425, and 450 MPa) at 750 °C for WAAM 740H superalloy after application of the designed post-heat treatment and compared with the performance of wrought Inconel 740H superalloy as shown in Figure 10b. Firstly, the applied stress (σ) and rupture time were collected from literature [26,27,28,29] for wrought Inconel 740H superalloy tested at 750 °C to evaluate the Larson-Miller (LM) parameter. From the plotted data available in the literature, excessive scatter in data points were observed for the LM parameters. A linear equation (σ = −0.05735 × LM + 1925) was fitted to the LM parameter vs. applied stress plot and further extrapolated to the high-stress regime where the tests were performed in this work. On superimposing the LM parameters evaluated from the test results for post-heat treated WAAM 740H superalloy, it was found to coincide closely with the fitted line, as can be seen in Figure 10b. This proves that the stress rupture performance of WAAM 740H superalloy is comparable with its wrought counterparts. However, there is still scope for improving this property for the WAAM 740H superalloy by replacing the homogenization step with HIP to promote gas pore closure.

The microstructural features after stress rupture tests were probed using SEM to understand the mechanism. No deleterious phases, such as the η phase, were observed after the stress rupture tests for the WAAM 740H superalloy. Near the fractured surface, cavities were observed along the grain boundaries, proving an intergranular mode of fracture, as shown in Figure 11b. Based on the reports available in the literature [27,30,31], grain boundary cavities form mainly in the γ’ denuded zone region close to the grain boundary. The mechanism for the formation of γ’ denuded zone in the 740H superalloy has been identified as the discontinuous coarsening of the γ’ phase that is catalyzed by grain boundary migration and sliding during creep [32]. In support of this mechanism, grain boundary migration was confirmed with the presence of serrated grain boundaries after stress rupture tests in this work, as shown in Figure 11c. It is worth mentioning that no significant coarsening of γ’ precipitates was observed after the stress rupture tests.

## 4. Conclusions

In this work, a crack-free build of Inconel 740H superalloy was fabricated successfully using wire arc additive manufacturing along with post-heat treatment design. The as-printed microstructure comprised of columnar γ grains as the matrix along with the Laves and (Nb, Ti) C as secondary phases. The anisotropy in grain structure increased from the bottom to the top portion of the build. The hardness of the middle portion was the highest, while the bottom was lowest due to the diffusion of iron from the mild steel substrate. For the post-heat treatment design, homogenization and aging temperatures were identified from the thermodynamic calculation, while the homogenization time was predicted using kinetic simulations. The optimum homogenization temperature and time were identified as 1200 °C for 2 h due to the complete recrystallization of microstructure along the build and dissolution of the Laves phase. The optimum aging time at 760 °C with peak hardness (~370 HV) was found to be 12 h. The designed post-heat treatment was significantly different from the standard heat treatment available for wrought Inconel 740H superalloy. The microstructure in peak aged condition consisted of thin and continuous as well as protruding lamellar M_23_C_6_ along the grain boundaries and grain interiors filled with the γ’ precipitates. The yield strength (655 MPa) and ductility (29.5%) were above the design targets (620 MPa and 20%) after post-heat treatment. The stress rupture performance of the WAAM Inconel 740H superalloy was at par with its wrought counterpart with an intergranular mode of fracture along with grain boundary migration and fractured MC carbides. The mechanical performance of this alloy can be further improved by replacing the homogenization step with hot isostatic pressing to promote gas pore closure, which is the scope for future work.

## Figures and Tables

**Table 1:** Composition (in wt.%) of the Inconel 740H wire used for WAAM as specified by the vendor.

Al	C	Co	Cr	Cu	Fe	Mn	Mo	Nb	Si	Ti	Ni
1.4	0.03	20.3	24.6	0.02	0.2	0.24	0.5	1.49	0.1	1.5	Bal.

## Disclaimer

This report was prepared as an account of work sponsored by an agency of the United States Government. Neither the United States Government nor any agency thereof, nor any of their employees, makes any warranty, express or implied, or assumes any legal liability or responsibility for the accuracy, completeness, or usefulness of any information, apparatus, product, or process disclosed, or represents that its use would not infringe privately owned rights. Reference herein to any specific commercial product, process, or service by trade name, trademark, manufacturer, or otherwise does not necessarily constitute or imply its endorsement, recommendation, or favoring by the United States Government or any agency thereof. The views and opinions of authors expressed herein do not necessarily state or reflect those of the United States Government or any agency thereof.
